# Supplementary material for: A chromosome-level assembly of the cat flea genome uncovers rampant gene duplication and genome size plasticity
Source: BMC Biol. 2020 Jun 19;18:70. doi: 10.1186/s12915-020-00802-7 (PMC7305587; doi:10.1186/s12915-020-00802-7)
Supplement: Supplementary file 1 — Additional file 1: Figure S1. Assessing assembly fragmentation, gene duplication and repeat elements within the C. felis assembly. (A) Evaluating assembly fragmentation via mapping of scaffolds shorter than 1 Mb (n = 3724) to scaffolds larger than 1 Mb (n = 9, “BIG9 scaffolds”). All but 2 short scaffolds mapped to a BIG9 scaffold at least once; confidence intervals are based on the probability of mapping to a single unique location. (B). Assessing the “genome completeness” of the C. felis full assembly and BIG9 scaffolds through comparison to eukaryote, arthropod and insect BUSCOs. (C) Tandem and proximal duplicate gene locations on BIG9 scaffold 1, (D) BIG9 scaffold 2, (E) BIG9 scaffold 3, (F) BIG9 scaffold 4, (G) BIG9 scaffold 5, (H) BIG9 scaffold 6, (I) BIG9 scaffold 7, (J) BIG9 scaffold 8, (K) BIG9 scaffold 9. (L) Duplications by proximity. Only true duplications (n = 2) are shown. Red bars (*) depict “dispersed” clusters that span multiple scaffolds. (M) Dispersed duplicate gene locations across BIG9 scaffolds. (N) Distribution across BIG9 scaffolds of C. felis proteins annotated as “DNA integration” (GO:0015074, see Additional file 2: Table S1. for specific accession numbers) and their relation to gene duplications. (O) Compilation of retroelements, DNA transposons and other repeat elements predicted across the BIG9 scaffolds. Overall totals are highlighted yellow. (P) tRNA gene abundances and (Q) codon usage/amino acid for select Holometabola. [file 12915_2020_802_MOESM1_ESM.pdf]

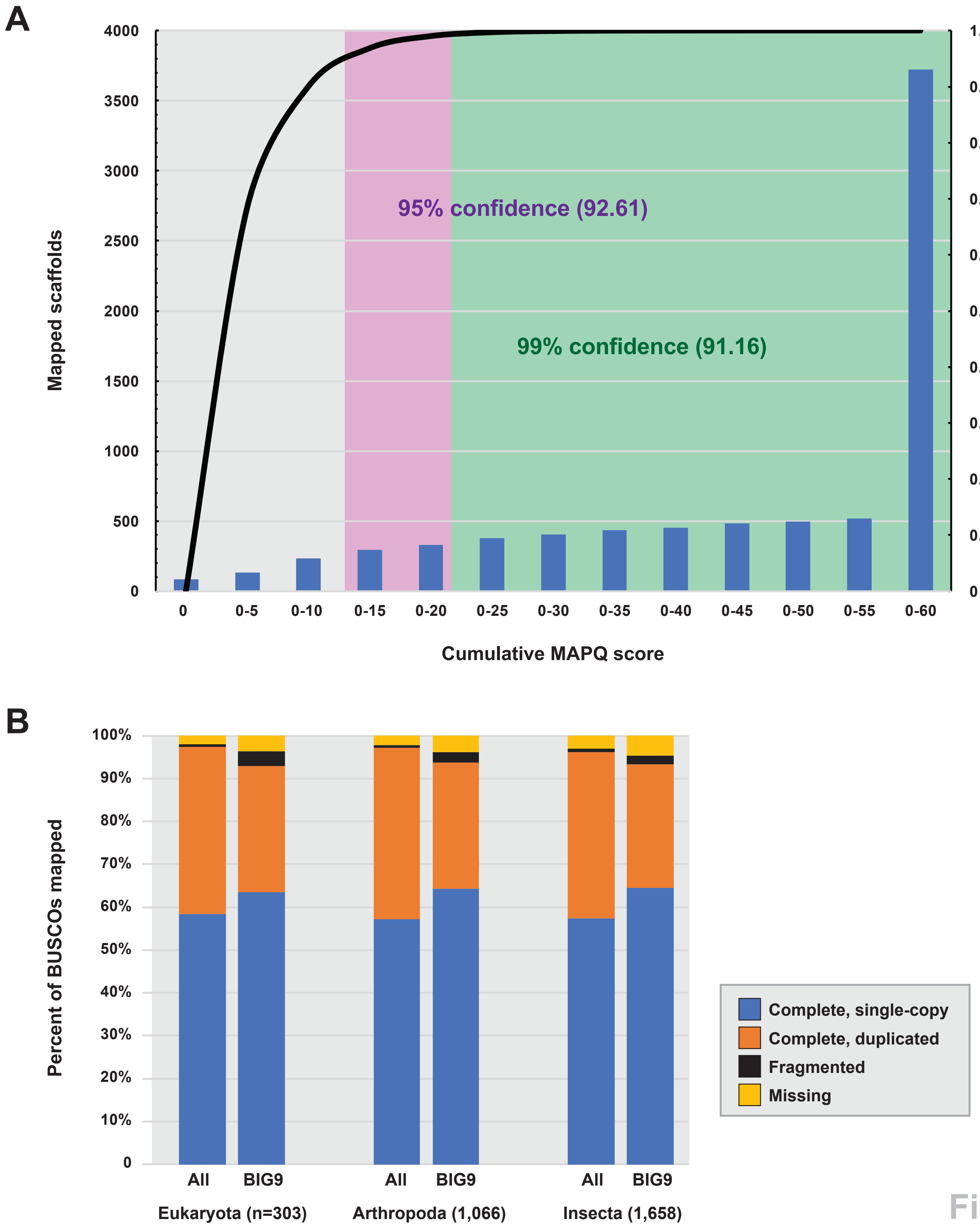
A
Mapped scaffolds
4000
3500
3000
2500
2000
1500
1000
500
0
Pr (mapped to single locus)
1.0
0.9
0.8
0.7
0.6
0.5
0.4
0.3
0.2
0.1
0
95% confidence (92.61)
99% confidence (91.16)
0
0-5
0-10
0-15
0-20
0-25
0-30
0-35
0-40
0-45
0-50
0-55
0-60
Cumulative MAPQ score
B
Percent of BUSCOs mapped
100%
90%
80%
70%
60%
50%
40%
30%
20%
10%
0
All
BIG9
All
BIG9
All
BIG9
Eukaryota (n=303)
Arthropoda (1,066)
Insecta (1,658)
Complete, single-copy
Complete, duplicated
Fragmented
Missing

Fig. S1

C **Scaffold 1 (NW_20538040.1)** **187,887,292 bp**

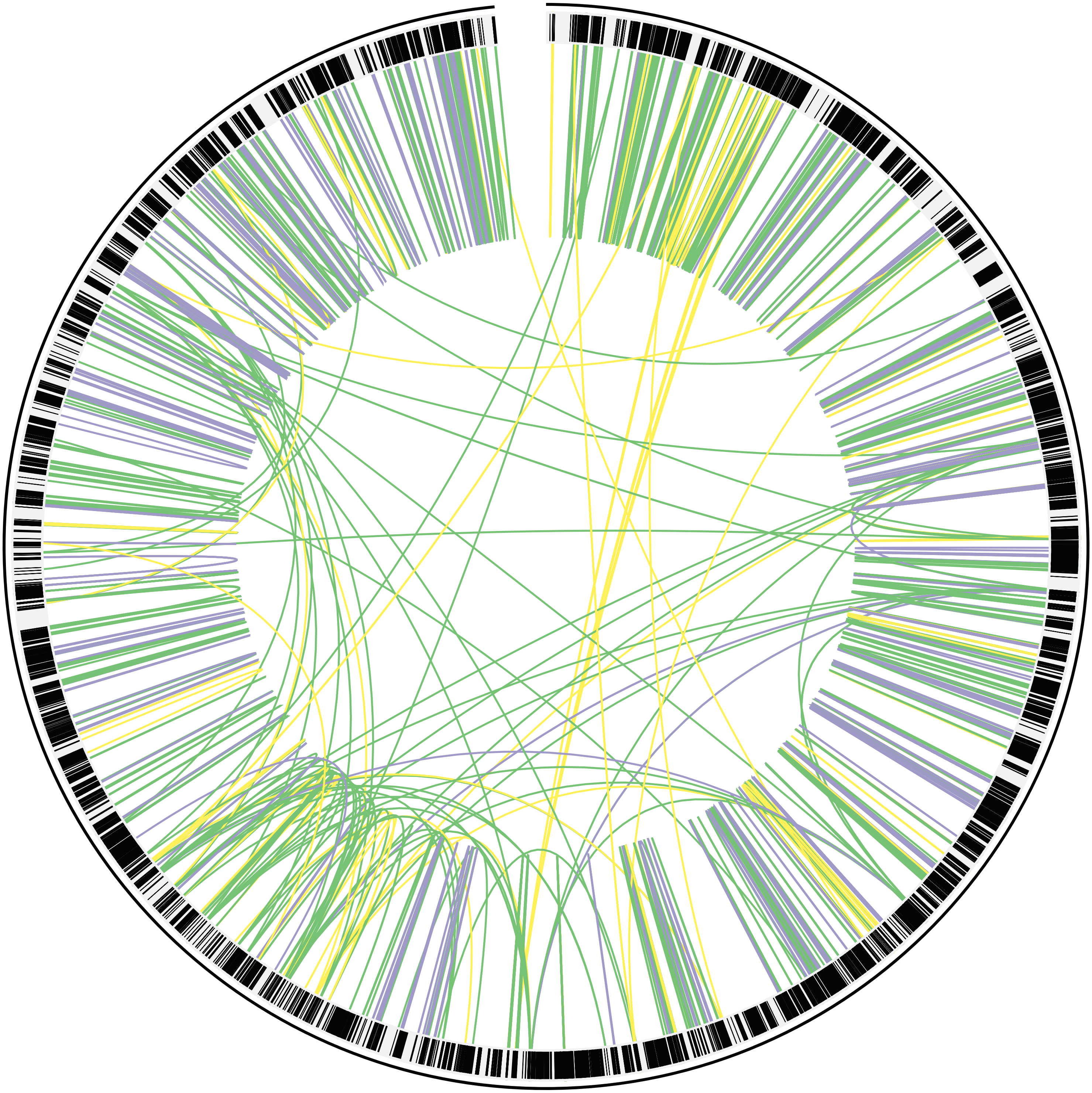

- 100 %ID
- 95-99 %ID
- 90-94 %ID

Scaffold	9	8	7	6	5	4	3	2	1
9 (NW_020539727)	304								
8 (NW_020539724)	2	360							
7 (NW_020539726)	4	2	444						
6 (NW_020537758)	3	7	18	573					
5 (NW_020537646)	22	15	44	34	909				
4 (NW_020539725)	6	19	23	14	42	1120			
3 (NW_020537324)	5	2	45	19	76	21	1135		
2 (NW_020536999)	4	13	48	32	64	43	68	1320	
1 (NW_020538040)	23	5	91	47	137	52	81	137	1314

Fig. S1

D **Scaffold 2 (NW_20536999.1)** **95,519,864 bp**

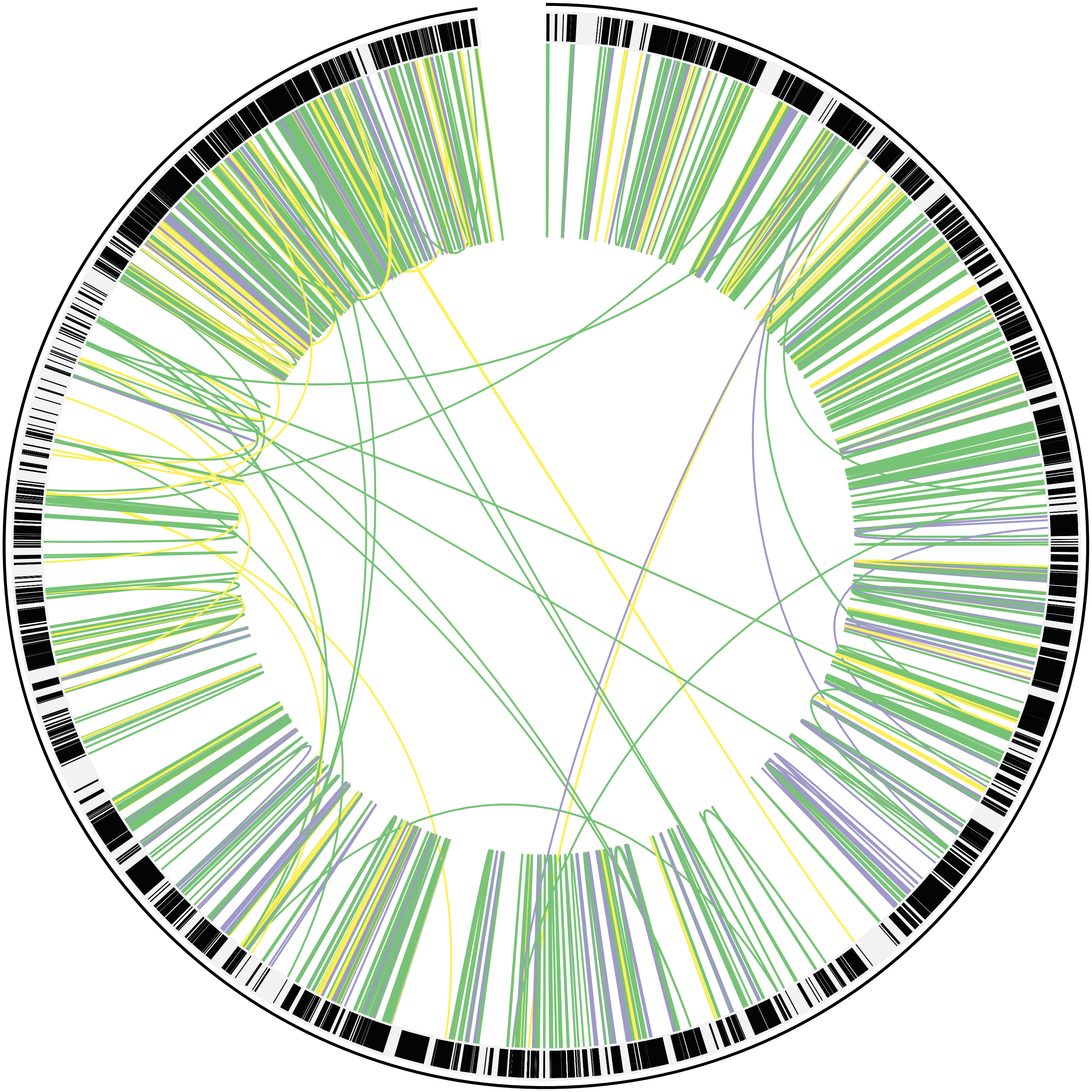

- 100 %ID
- 95-99 %ID
- 90-94 %ID

Scaffold	9	8	7	6	5	4	3	2	1
9 (NW_020539727)	304								
8 (NW_020539724)	2	360							
7 (NW_020539726)	4	2	444						
6 (NW_020537758)	3	7	18	573					
5 (NW_020537646)	22	15	44	34	909				
4 (NW_020539725)	6	19	23	14	42	1120			
3 (NW_020537324)	5	2	45	19	76	21	1135		
2 (NW_020536999)	4	13	48	32	64	43	68	1320	
1 (NW_020538040)	23	5	91	47	137	52	81	137	1314

Fig. S1

E **Scaffold 3 (NW_20537324.1)** **87,719,082 bp**

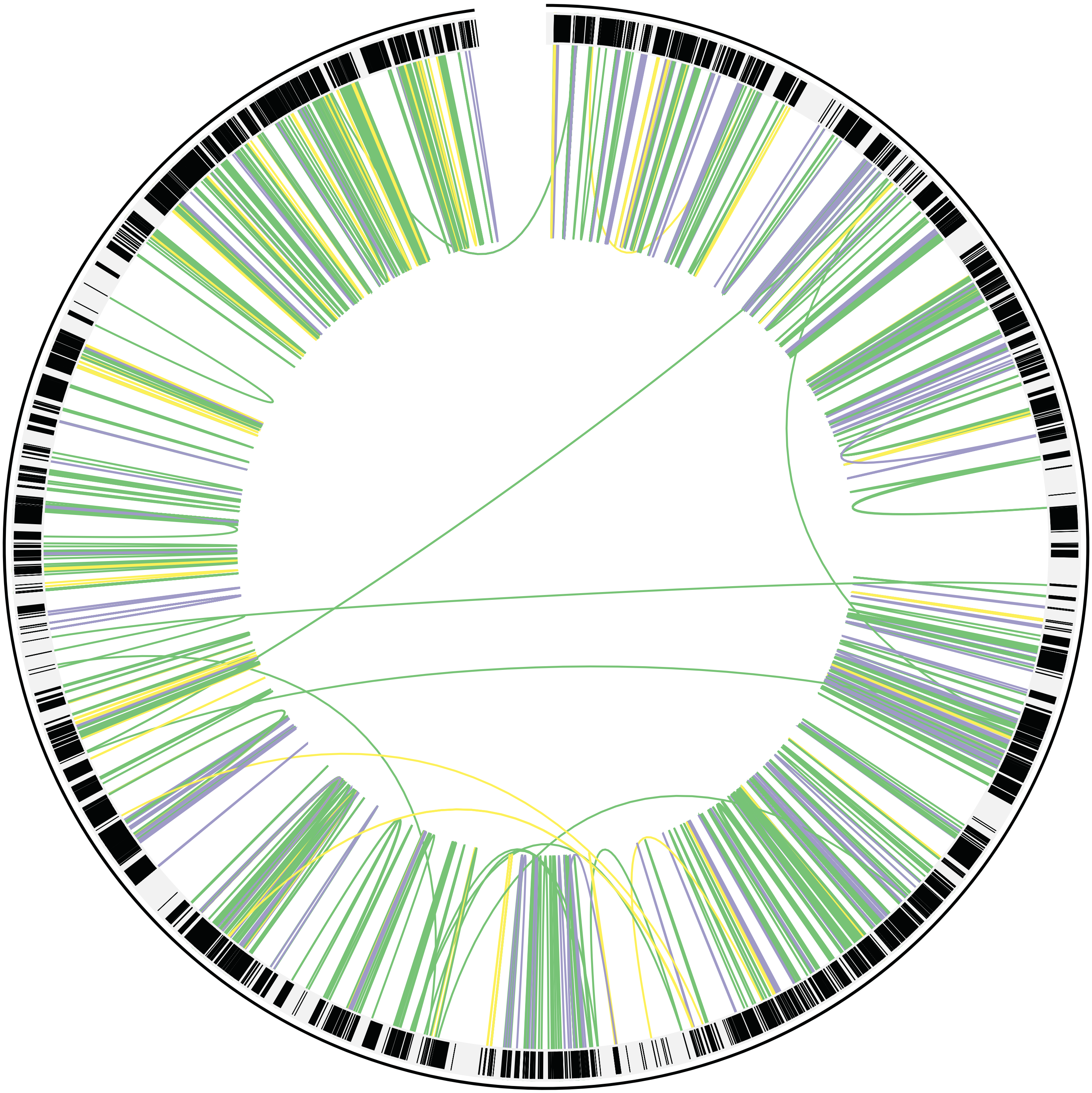

- 100 %ID
- 95-99 %ID
- 90-94 %ID

Scaffold	9	8	7	6	5	4	3	2	1
9 (NW_020539727)	304								
8 (NW_020539724)	2	360							
7 (NW_020539726)	4	2	444						
6 (NW_020537758)	3	7	18	573					
5 (NW_020537646)	22	15	44	34	909				
4 (NW_020539725)	6	19	23	14	42	1120			
3 (NW_020537324)	5	2	45	19	76	21	1135		
2 (NW_020536999)	4	13	48	32	64	43	68	1320	
1 (NW_020538040)	23	5	91	47	137	52	81	137	1314

Fig. S1

F **Scaffold 4 (NW_020539725.1)** **71,713,785 bp**

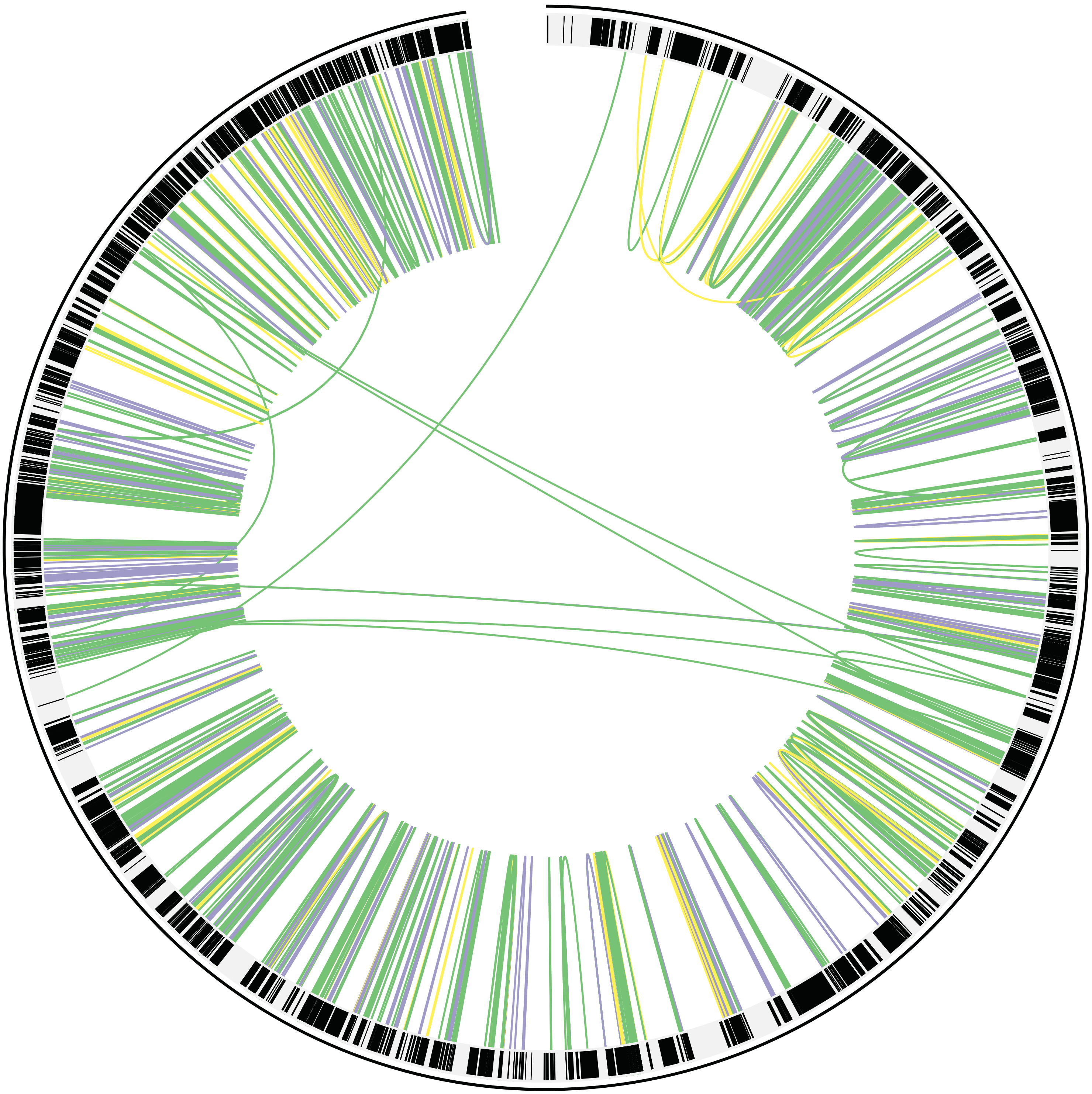

- 100 %ID
- 95-99 %ID
- 90-94 %ID

Scaffold	9	8	7	6	5	4	3	2	1
9 (NW_020539727)	304								
8 (NW_020539724)	2	360							
7 (NW_020539726)	4	2	444						
6 (NW_020537758)	3	7	18	573					
5 (NW_020537646)	22	15	44	34	909				
4 (NW_020539725)	6	19	23	14	42	1120			
3 (NW_020537324)	5	2	45	19	76	21	1135		
2 (NW_020536999)	4	13	48	32	64	43	68	1320	
1 (NW_020538040)	23	5	91	47	137	52	81	137	1314

Fig. S1

G **Scaffold 5 (NW_020537646.1)** **61,547,676 bp**

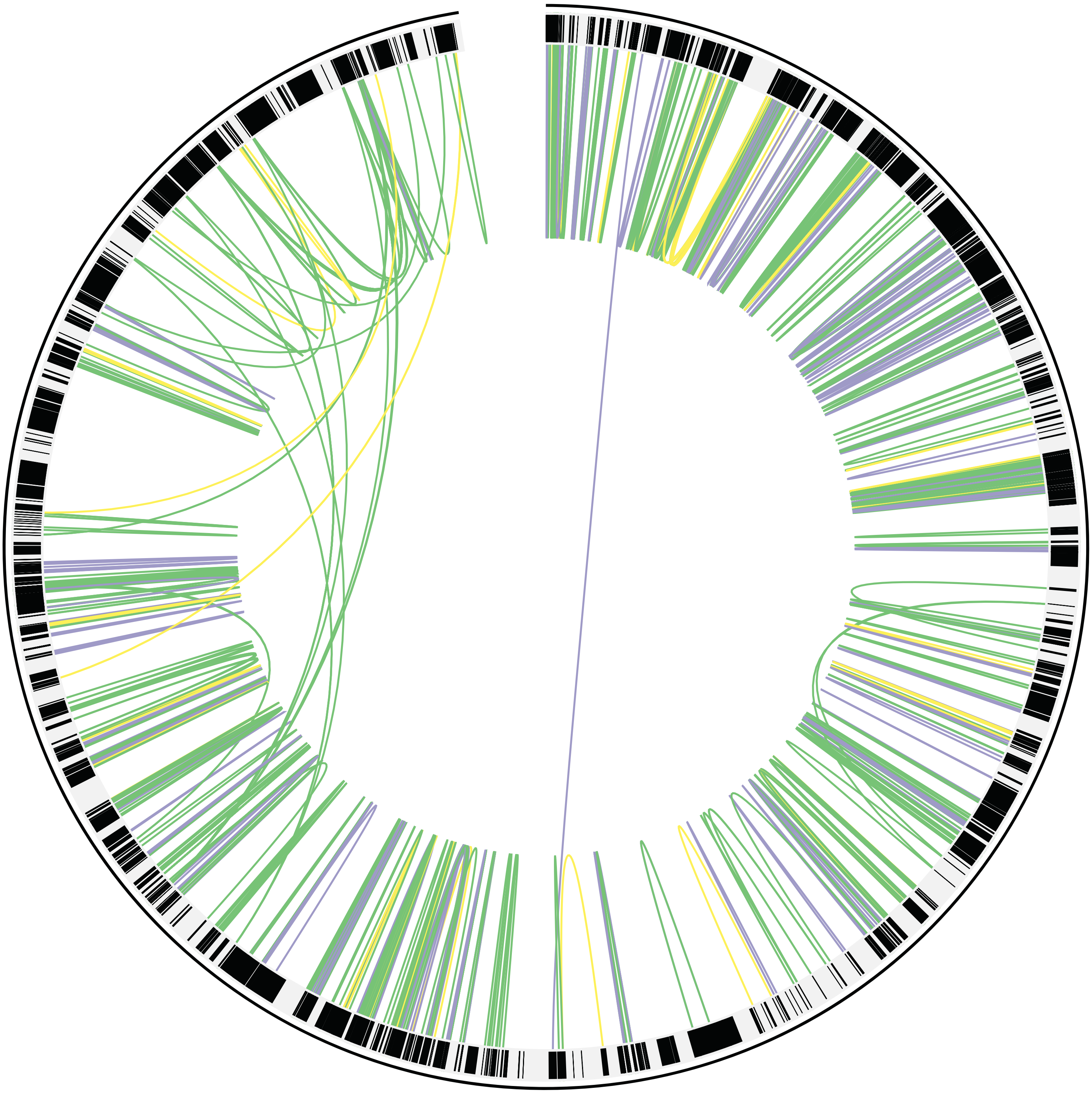

- 100 %ID
- 95-99 %ID
- 90-94 %ID

Scaffold	9	8	7	6	5	4	3	2	1
9 (NW_020539727)	304								
8 (NW_020539724)	2	360							
7 (NW_020539726)	4	2	444						
6 (NW_020537758)	3	7	18	573					
5 (NW_020537646)	22	15	44	34	909				
4 (NW_020539725)	6	19	23	14	42	1120			
3 (NW_020537324)	5	2	45	19	76	21	1135		
2 (NW_020536999)	4	13	48	32	64	43	68	1320	
1 (NW_020538040)	23	5	91	47	137	52	81	137	1314

Fig. S1

H **Scaffold 6 (NW_020537758.1)** **59,836,375 bp**

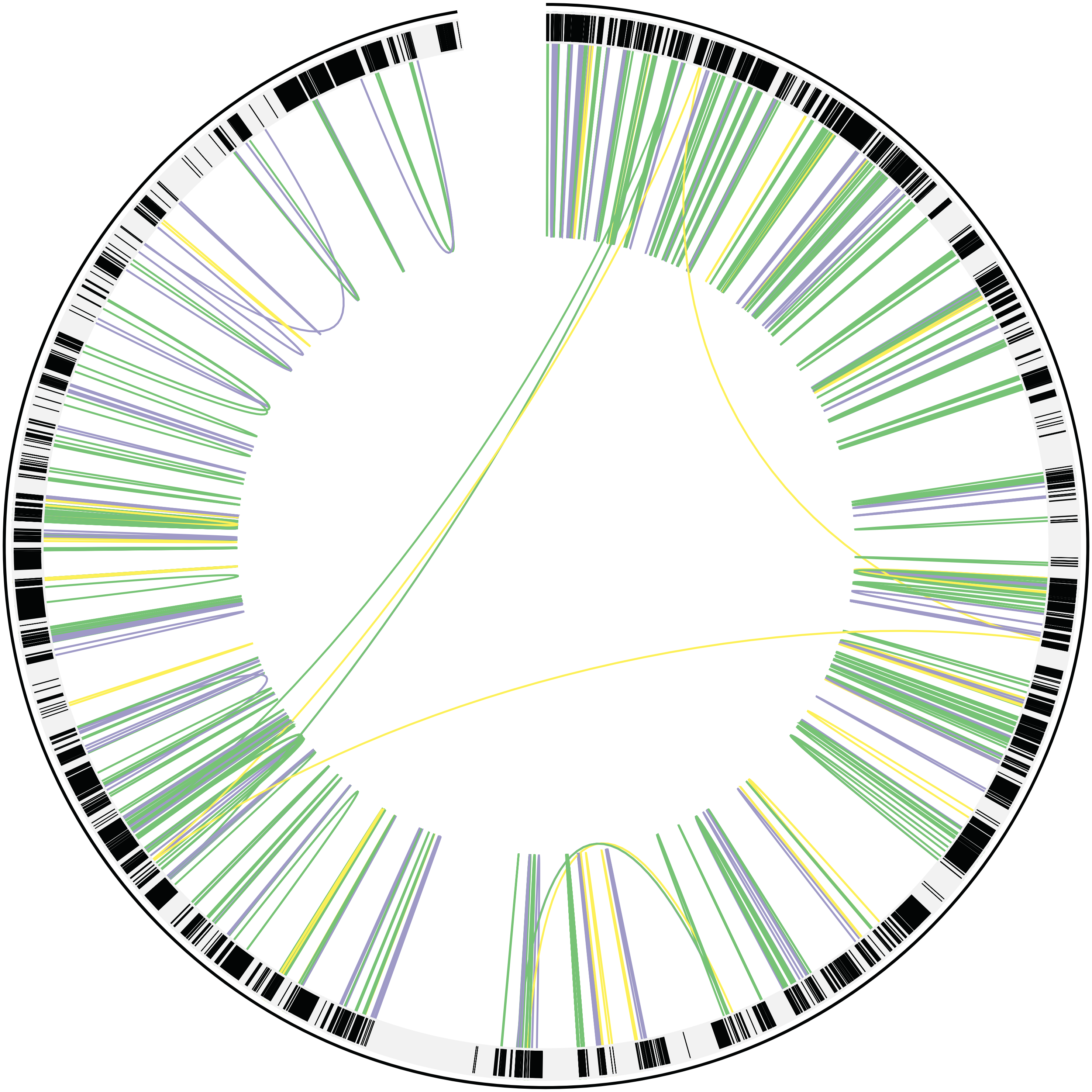

Scaffold	9	8	7	6	5	4	3	2	1
9 (NW_020539727)	304								
8 (NW_020539724)	2	360							
7 (NW_020539726)	4	2	444						
6 (NW_020537758)	3	7	18	573					
5 (NW_020537646)	22	15	44	34	909				
4 (NW_020539725)	6	19	23	14	42	1120			
3 (NW_020537324)	5	2	45	19	76	21	1135		
2 (NW_020536999)	4	13	48	32	64	43	68	1320	
1 (NW_020538040)	23	5	91	47	137	52	81	137	1314

I

Scaffold 7 (NW_020539726.1) **46,735,595 bp**

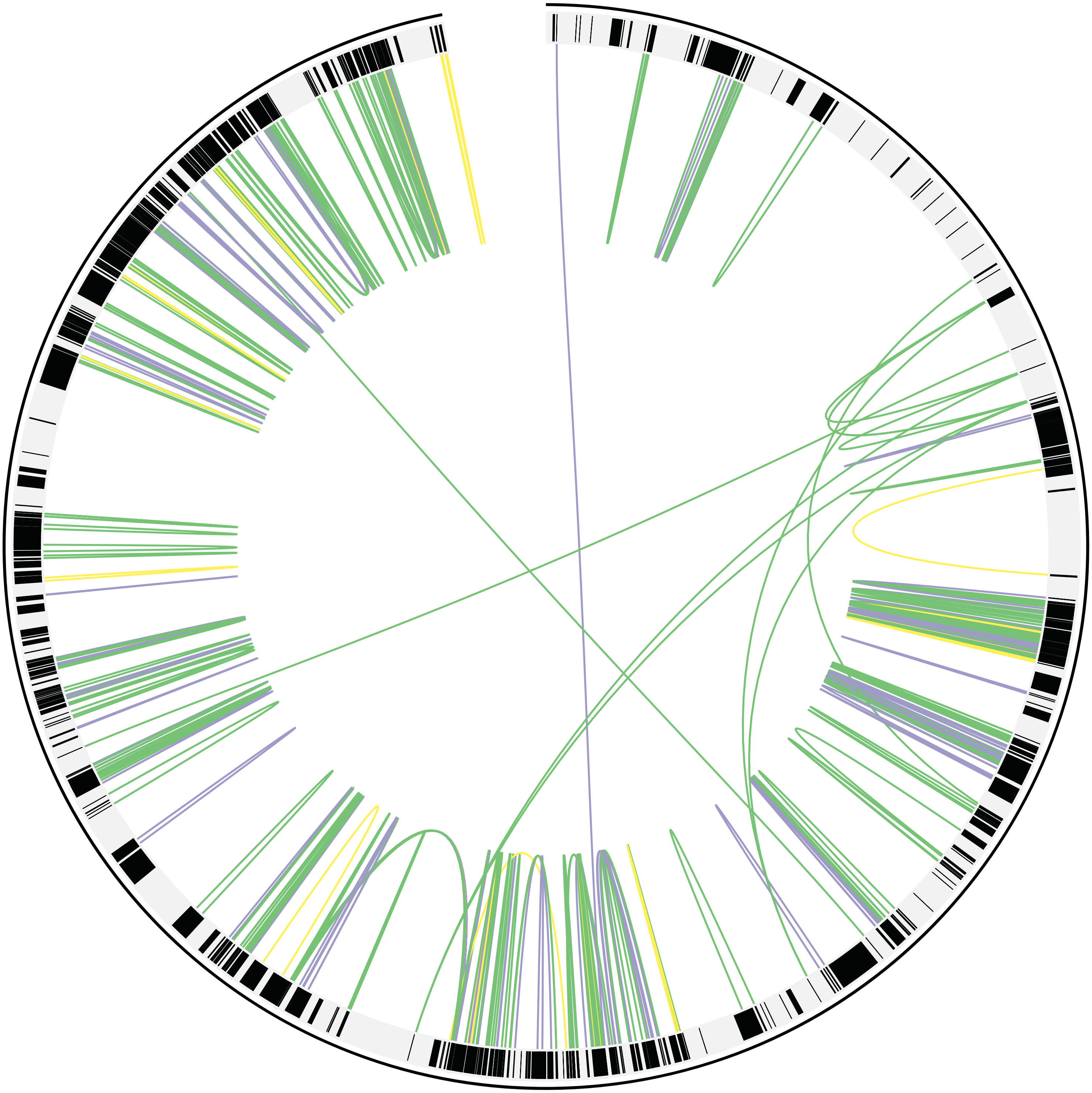

100 %ID

95-99 %ID

90-94 %ID

Scaffold	9	8	7	6	5	4	3	2	1
9 (NW_020539727)	304								
8 (NW_020539724)	2	360							
7 (NW_020539726)	4	2	444						
6 (NW_020537758)	3	7	18	573					
5 (NW_020537646)	22	15	44	34	909				
4 (NW_020539725)	6	19	23	14	42	1120			
3 (NW_020537324)	5	2	45	19	76	21	1135		
2 (NW_020536999)	4	13	48	32	64	43	68	1320	
1 (NW_020538040)	23	5	91	47	137	52	81	137	1314

Fig. S1

J **Scaffold 8 (NW_020539724.1)** **28,733,183 bp**

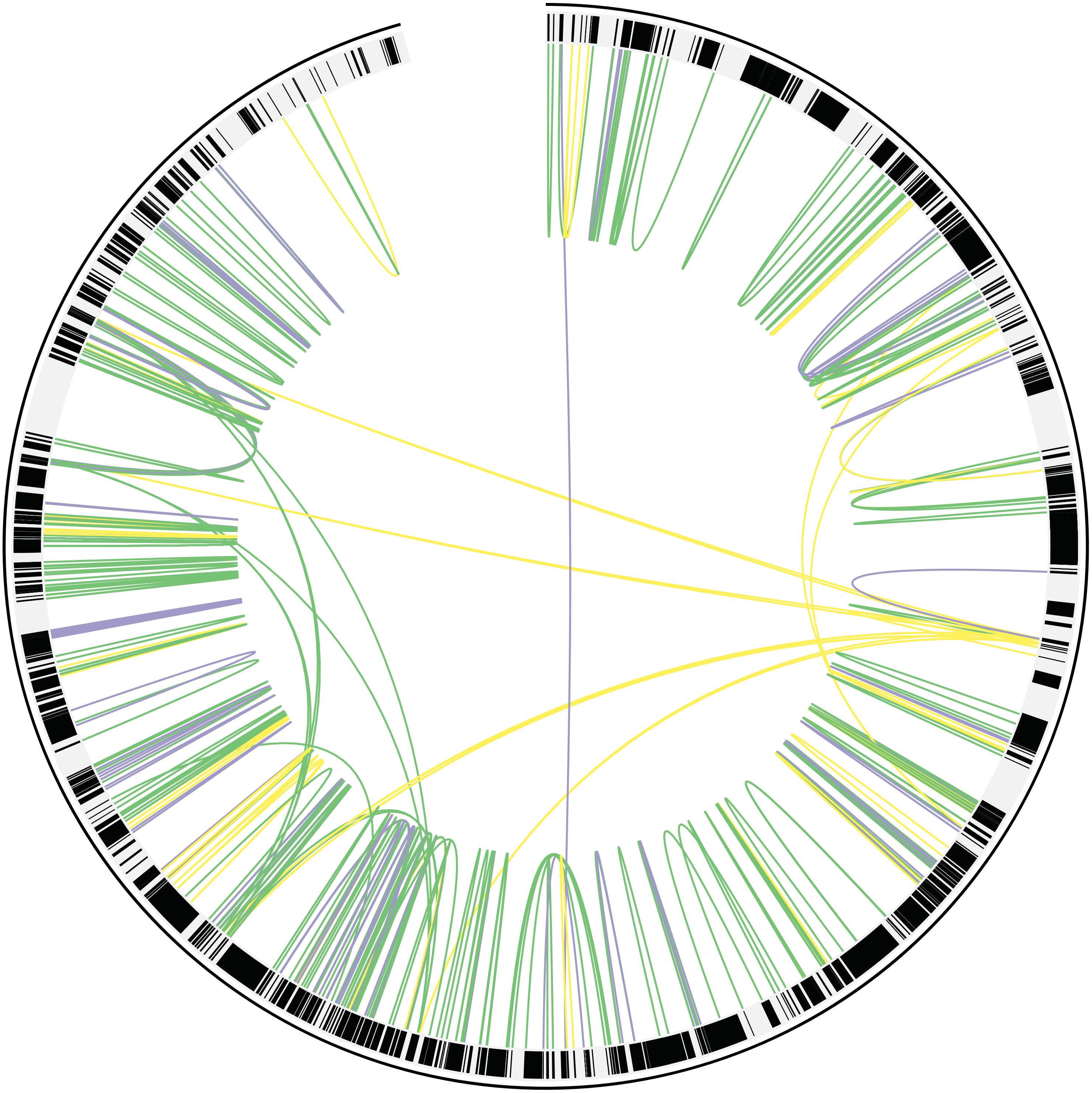

- 100 %ID
- 95-99 %ID
- 90-94 %ID

Scaffold	9	8	7	6	5	4	3	2	1
9 (NW_020539727)	304								
8 (NW_020539724)	2	360							
7 (NW_020539726)	4	2	444						
6 (NW_020537758)	3	7	18	573					
5 (NW_020537646)	22	15	44	34	909				
4 (NW_020539725)	6	19	23	14	42	1120			
3 (NW_020537324)	5	2	45	19	76	21	1135		
2 (NW_020536999)	4	13	48	32	64	43	68	1320	
1 (NW_020538040)	23	5	91	47	137	52	81	137	1314

Fig. S1

K **Scaffold 9 (NW_020539727.1)** **25,564,690 bp**

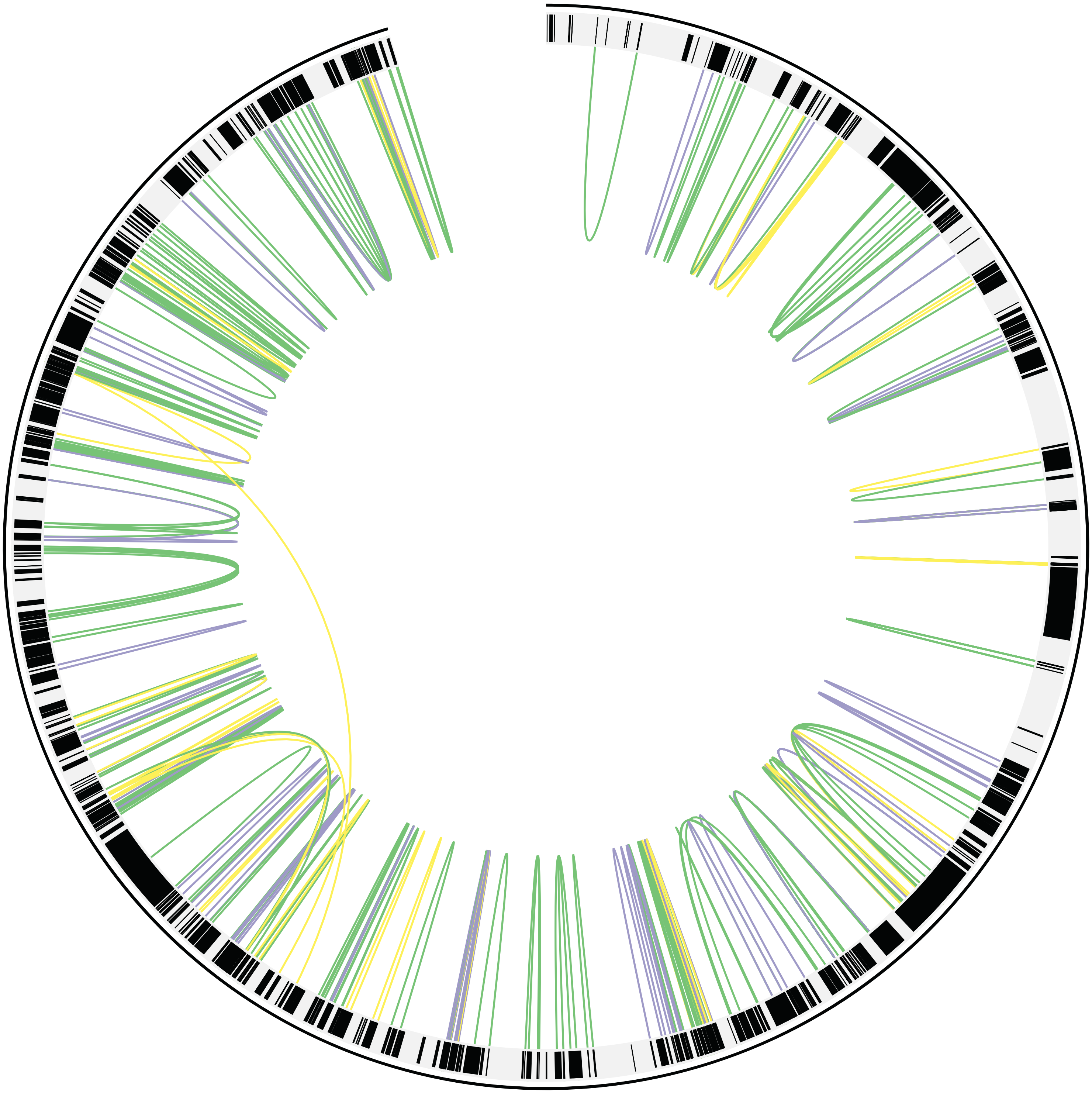

- 100 %ID
- 95-99 %ID
- 90-94 %ID

Scaffold	9	8	7	6	5	4	3	2	1
9 (NW_020539727)	304								
8 (NW_020539724)	2	360							
7 (NW_020539726)	4	2	444						
6 (NW_020537758)	3	7	18	573					
5 (NW_020537646)	22	15	44	34	909				
4 (NW_020539725)	6	19	23	14	42	1120			
3 (NW_020537324)	5	2	45	19	76	21	1135		
2 (NW_020536999)	4	13	48	32	64	43	68	1320	
1 (NW_020538040)	23	5	91	47	137	52	81	137	1314

Fig. S1

L

Fig. S1

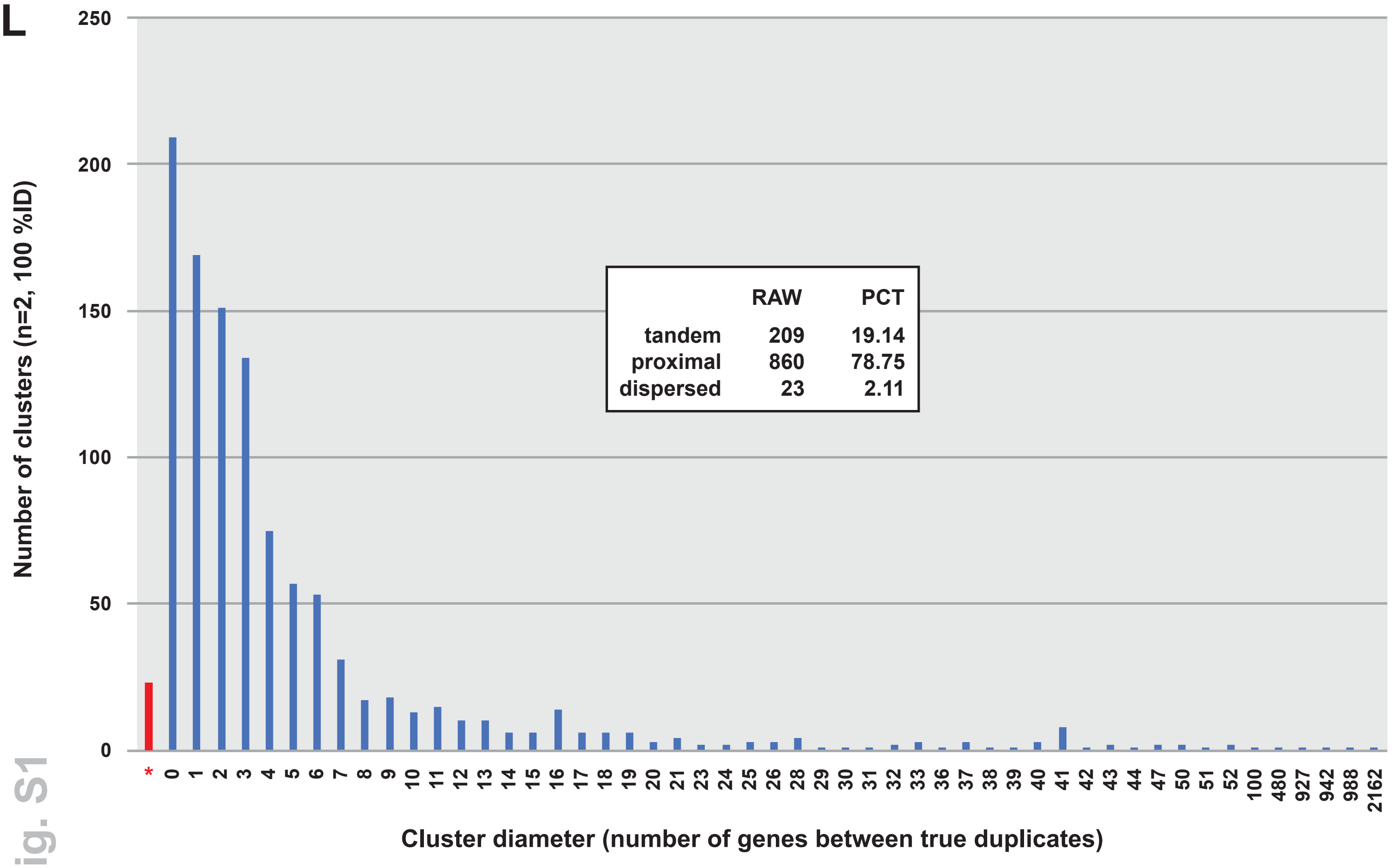

Number of clusters (n=2, 100 %ID)
250
200
150
100
50
0
RAW PCT
tandem 209 19.14
proximal 860 78.75
dispersed 23 2.11
* 0 1 2 3 4 5 6 7 8 9 10 11 12 13 14 15 16 17 18 19 20 21 23 24 25 26 28 29 30 31 32 33 36 37 38 39 40 41 42 43 44 47 50 51 52 100 480 927 942 988 2162
Cluster diameter (number of genes between true duplicates)

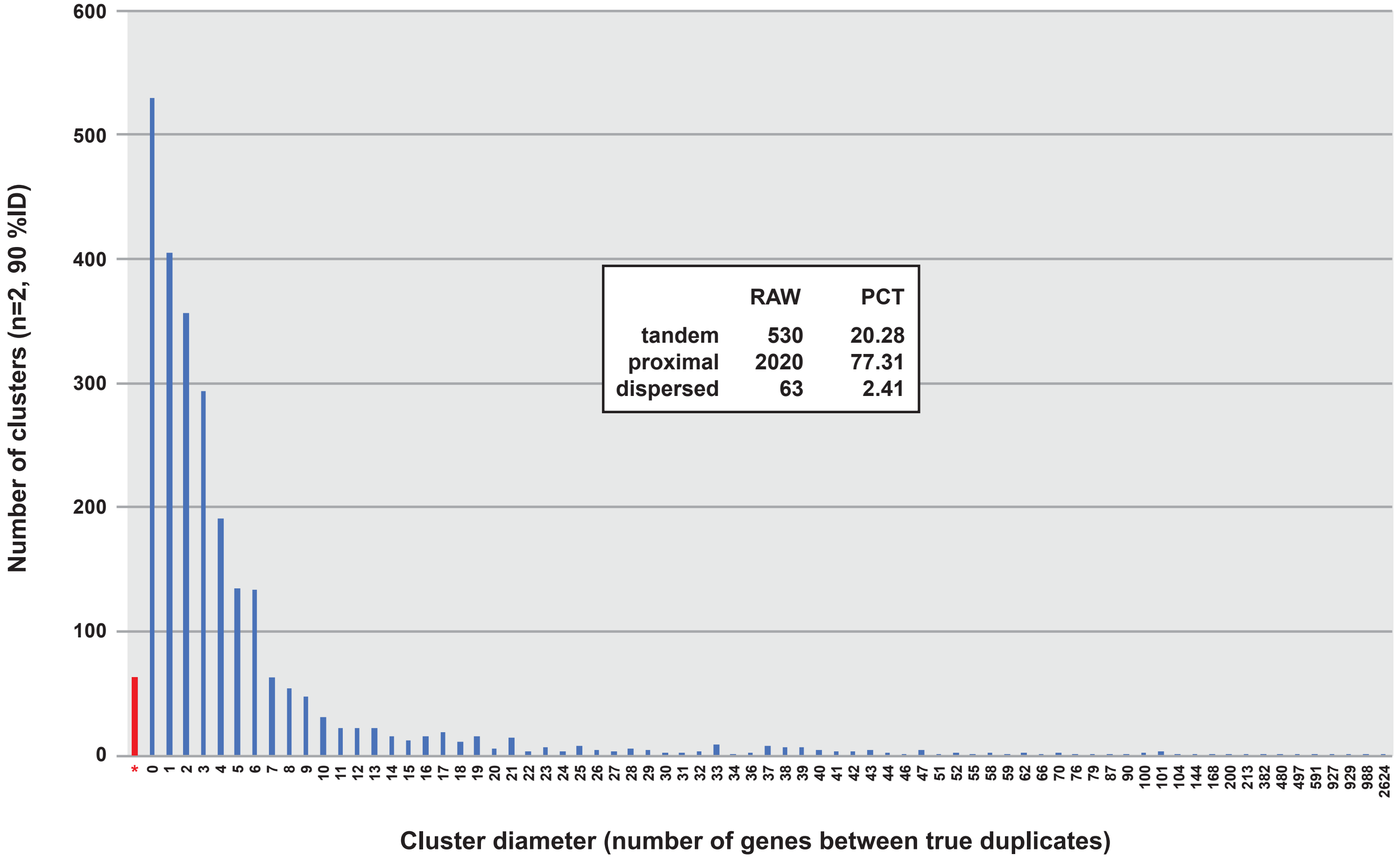

Number of clusters (n=2, 90 %ID)
600
500
400
300
200
100
0
RAW PCT
tandem 530 20.28
proximal 2020 77.31
dispersed 63 2.41
* 0 1 2 3 4 5 6 7 8 9 10 11 12 13 14 15 16 17 18 19 20 21 22 23 24 25 26 27 28 29 30 31 32 33 34 36 37 38 39 40 41 42 43 44 46 47 51 52 55 58 59 62 66 70 76 79 87 90 100 101 104 144 168 200 213 382 480 497 591 927 929 988 2624
Cluster diameter (number of genes between true duplicates)

M

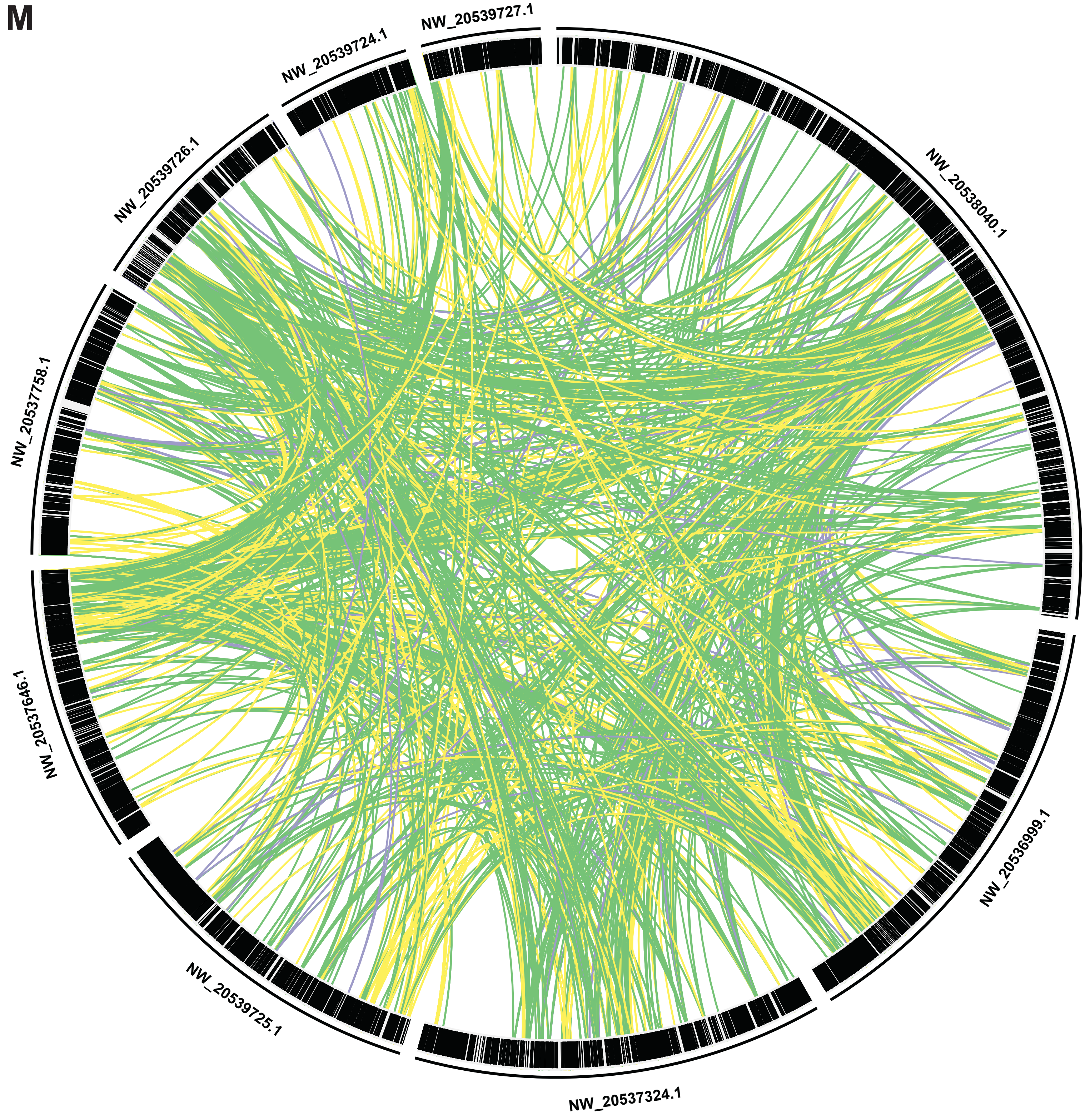

100 %ID

95-99 %ID

90-94 %ID

Scaffold	9	8	7	6	5	4	3	2	1
9 (NW_020539727)	304								
8 (NW_020539724)	2	360							
7 (NW_020539726)	4	2	444						
6 (NW_020537758)	3	7	18	573					
5 (NW_020537646)	22	15	44	34	909				
4 (NW_020539725)	6	19	23	14	42	1120			
3 (NW_020537324)	5	2	45	19	76	21	1135		
2 (NW_020536999)	4	13	48	32	64	43	68	1320	
1 (NW_020538040)	23	5	91	47	137	52	81	137	1314

Fig. S1

N

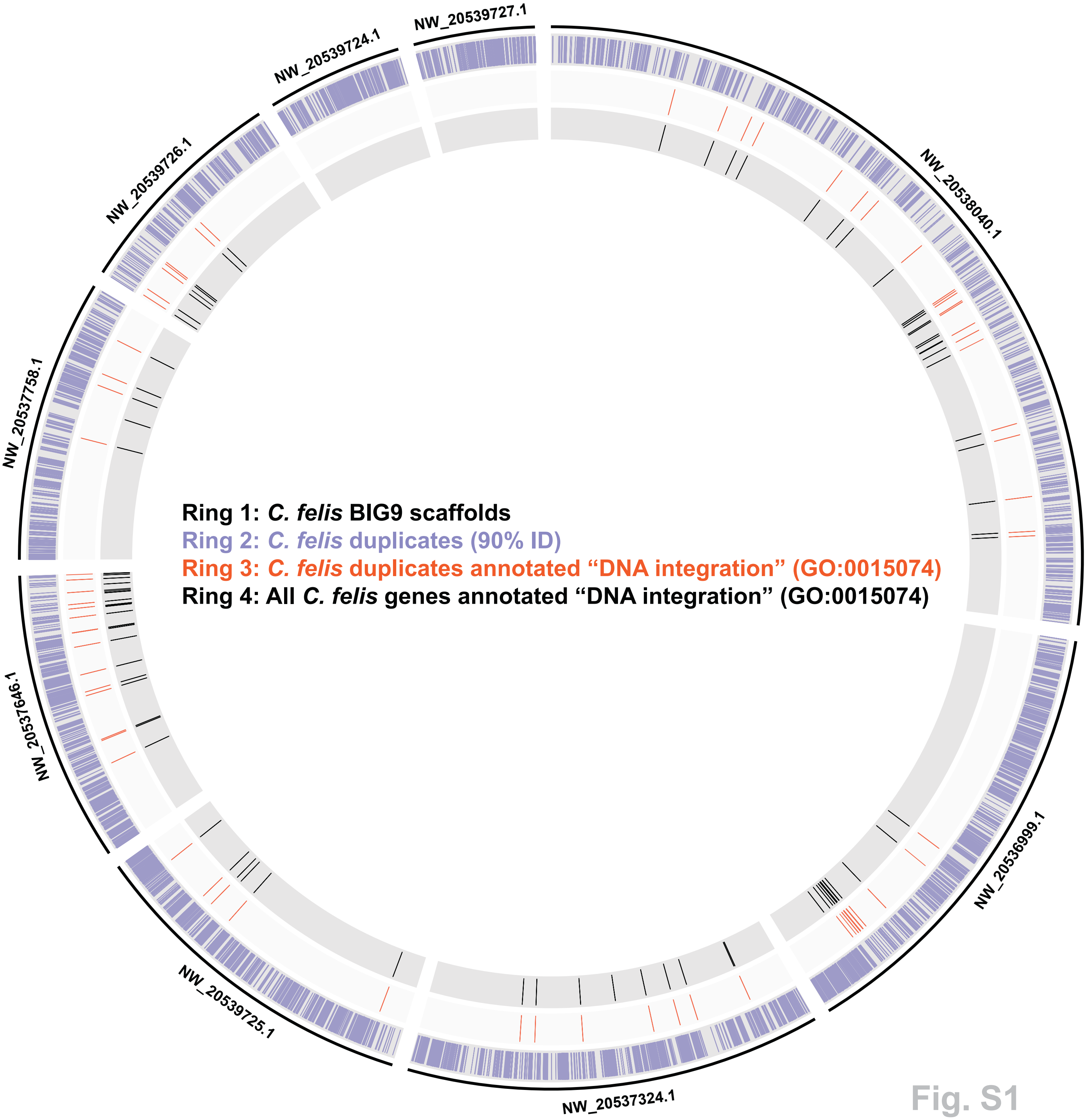
NW_20539727.1
NW_20539724.1
NW_20539726.1
NW_20538040.1
NW_20537758.1
NW_20537646.1
NW_20536999.1
NW_20539725.1
NW_20537324.1
Ring 1: *C. felis* BIG9 scaffolds
Ring 2: *C. felis* duplicates (90% ID)
Ring 3: *C. felis* duplicates annotated "DNA integration" (GO:0015074)
Ring 4: All *C. felis* genes annotated "DNA integration" (GO:0015074)

Fig. S1

O

Superfamily	Total no.	Total Length	% of BIG9
Retroelements	61,071	12,619,486	1.9295
SINEs	336	26,535	0.0041
LINEs	34,965	8,515,826	1.302
Penelope	3,086	499,380	0.0764
CRE/SLACS	2	162	0.0000
L2/CR1/Rex	10,015	1,081,344	0.1653
R2/R4/NeSL	491	177,375	0.0271
L1/CIN4	445	61,750	0.0094
RTEs	713	133,953	0.0205
Other LINEs	20,213	6,561,862	1.0033
LTRs	25,770	4,077,125	0.6234
Unclassifed LTR	94	5,062	0.0008
Bel/Pao	5,675	989,527	0.1513
Ty1/Copia	991	137,555	0.0210
Gypsy/DIRS1	19,010	2,944,981	0.4503
DNA transposons	182,844	24,537,653	3.752
Unclassified DNA transposons	91,977	12,373,802	1.8919
hAT	9,939	960,540	0.1469
IS630-Tc1-Mariner	36,131	6,148,685	0.9401
En-Spm	80	5,537	0.0008
MuDR	3,801	352,443	0.0539
PiggyBac	1,934	302,651	0.0463
Tourist/Harbinger/PIF	1,935	156,040	0.0239
Zator	15,858	1,770,968	0.2708
Sola	408	62,065	0.0095
Other DNA transposons	20,781	2,404,922	0.3677
Rolling Circles	29,853	4,381,462	0.6699
Unclassified Repeats	1,866	232,894	0.0356
rRNA/tRNA	3,703	809,578	0.1238
Satellites	206	34,766	0.0053
Simple Repeats	42,5231	19,488,965	2.9798
Low Complexity	62,484	3,137,132	0.4797
Total Repeat Elements:	767,258	65,241,936	9.975

Fig. S1

P

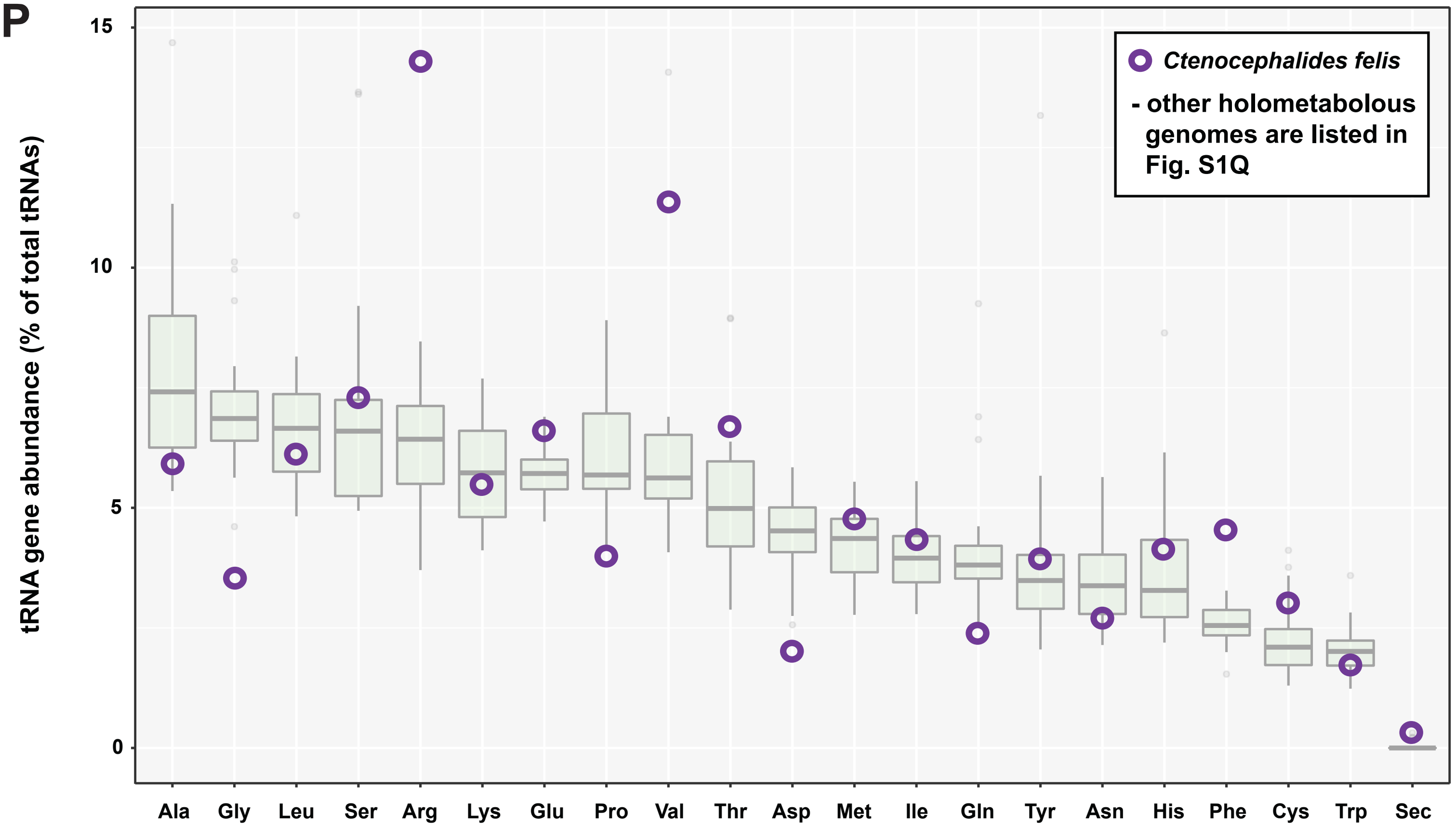

Ctenocephalides felis
- other holometabolous genomes are listed in Fig. S1Q
tRNA gene abundance (% of total tRNAs)
15
10
5
0
Ala
Gly
Leu
Ser
Arg
Lys
Glu
Pro
Val
Thr
Asp
Met
Ile
Gln
Tyr
Asn
His
Phe
Cys
Trp
Sec

Q

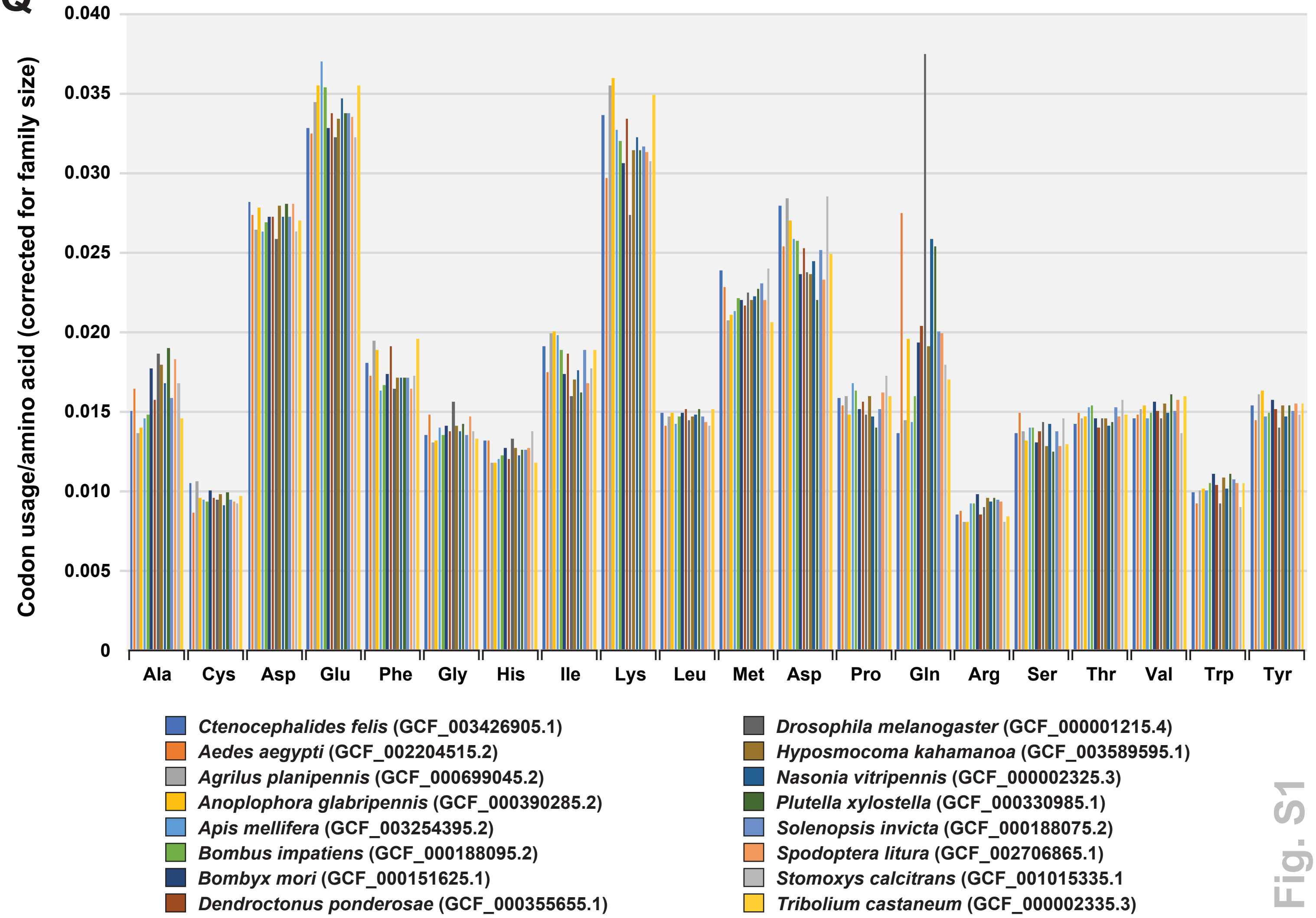

Codon usage/amino acid (corrected for family size)
0.040
0.035
0.030
0.025
0.020
0.015
0.010
0.005
0
Ala
Cys
Asp
Glu
Phe
Gly
His
Ile
Lys
Leu
Met
Asp
Pro
Gln
Arg
Ser
Thr
Val
Trp
Tyr
Ctenocephalides felis (GCF_003426905.1)
Aedes aegypti (GCF_002204515.2)
Agrilus planipennis (GCF_000699045.2)
Anoplophora glabripennis (GCF_000390285.2)
Apis mellifera (GCF_003254395.2)
Bombus impatiens (GCF_000188095.2)
Bombyx mori (GCF_000151625.1)
Dendroctonus ponderosae (GCF_000355655.1)
Drosophila melanogaster (GCF_000001215.4)
Hyposmocoma kahamanoa (GCF_003589595.1)
Nasonia vitripennis (GCF_000002325.3)
Plutella xylostella (GCF_000330985.1)
Solenopsis invicta (GCF_000188075.2)
Spodoptera litura (GCF_002706865.1)
Stomoxys calcitrans (GCF_001015335.1
Tribolium castaneum (GCF_000002335.3)

Fig. S1
