# Supplementary figures and images for: A chromosome-level assembly of the cat flea genome uncovers rampant gene duplication and genome size plasticity
Source: BMC Biol. 2020 Jun 19;18:70. doi: 10.1186/s12915-020-00802-7 (PMC7305587; doi:10.1186/s12915-020-00802-7)

**A**

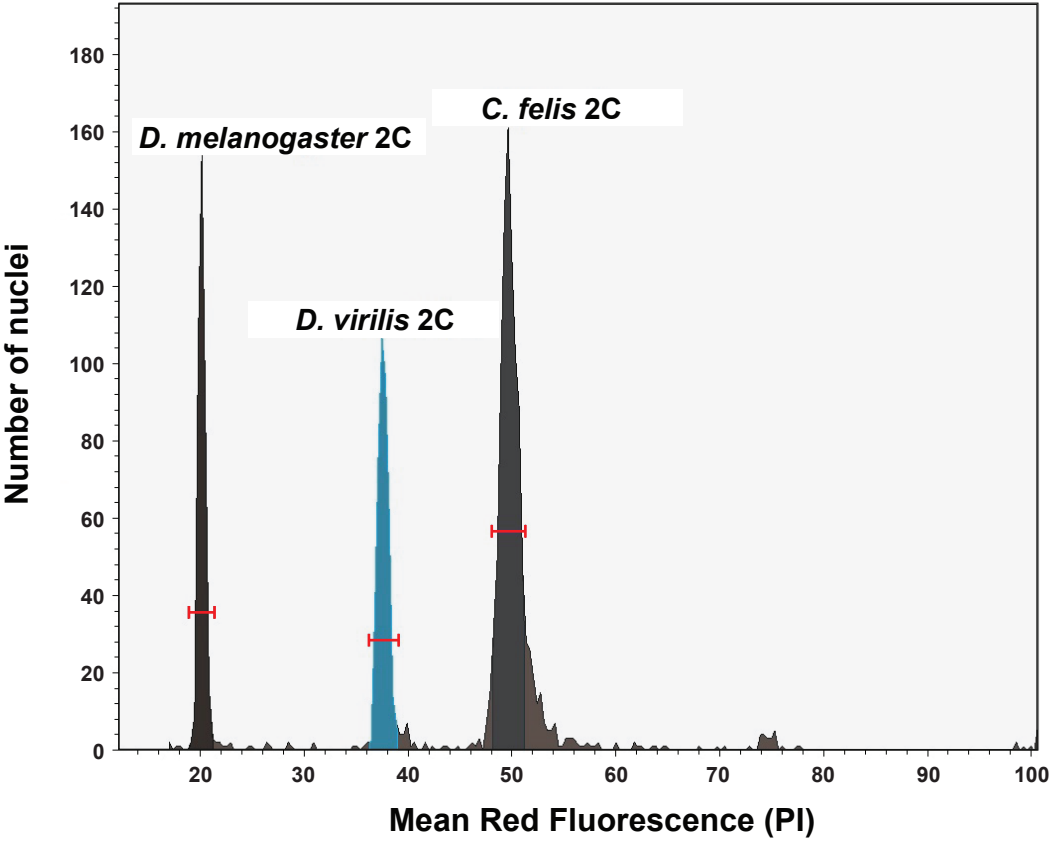

**B**

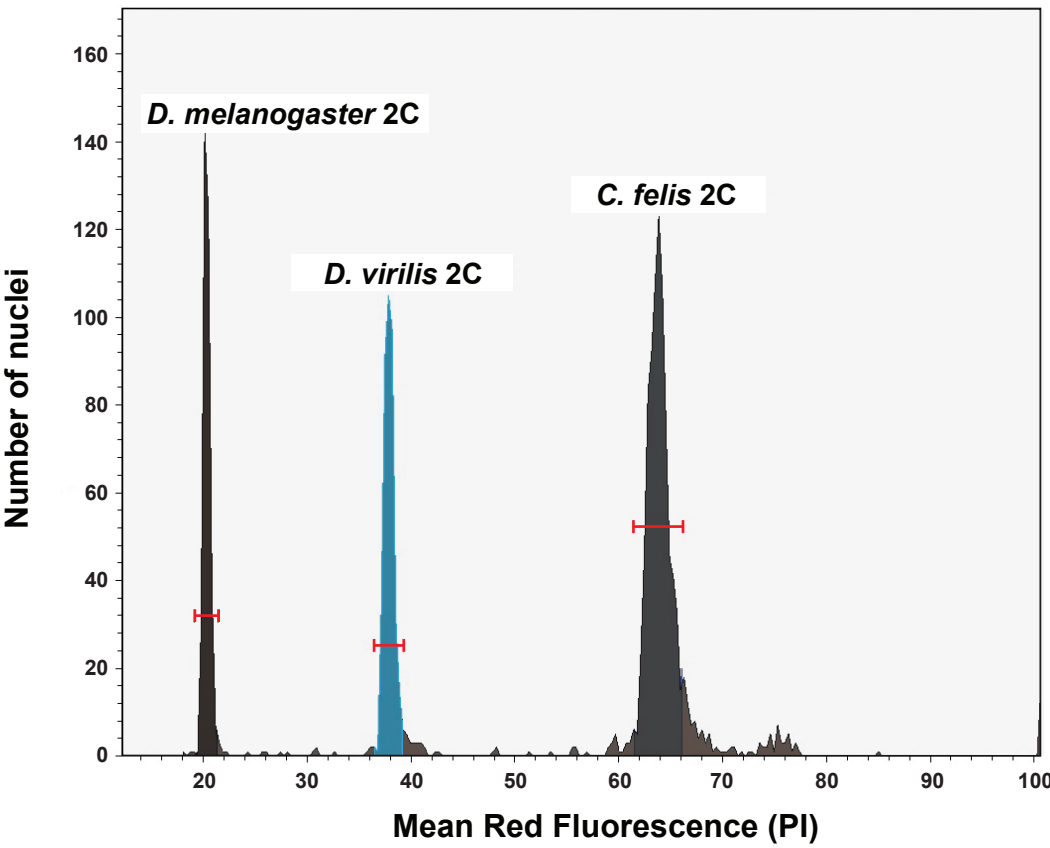

Fig. S2

Supplement: Supplementary file 3 — Additional file 3: Figure S2. Representative histograms produced by flow cytometry showing the peak positions of the 2C nuclei of Drosophila melanogaster (left) and D. virilis (center) female standards, and individual C. felis females (right) from the sequenced EL strain. (A) A 434 Mb flea. (B) A 553 Mb flea. All peaks have CV < 1.5 and > 500 nuclei under the statistical gates (red lines spanning the 2C peaks). [file 12915_2020_802_MOESM3_ESM.pdf]
