# Supplementary material for: A chromosome-level assembly of the cat flea genome uncovers rampant gene duplication and genome size plasticity
Source: BMC Biol. 2020 Jun 19;18:70. doi: 10.1186/s12915-020-00802-7 (PMC7305587; doi:10.1186/s12915-020-00802-7)
Supplement: Supplementary file 4 — Additional file 4: Figure S3. Phylogenomics analysis of select Holometabola. (A) Assessment of holometabolan accessory genomes. (B) Top: Identification of conserved protein families present in select taxa from each holometabolan order but absent from C. felis. Bottom: Protein families conserved across all sequenced holometabolan genomes except C. felis (see Additional file 5: Table S2). Four assemblies were identified as particularly patchy (Oryctes borbonicus, Operophtera brumata, Heliothis virescens, and Plutella xylostella) and 100% conservation (“perfect”) was also relaxed to exclude these taxa. Inset, redrawn phylogeny estimation of Holometabola [2]. [file 12915_2020_802_MOESM4_ESM.pdf]

Fig. S3

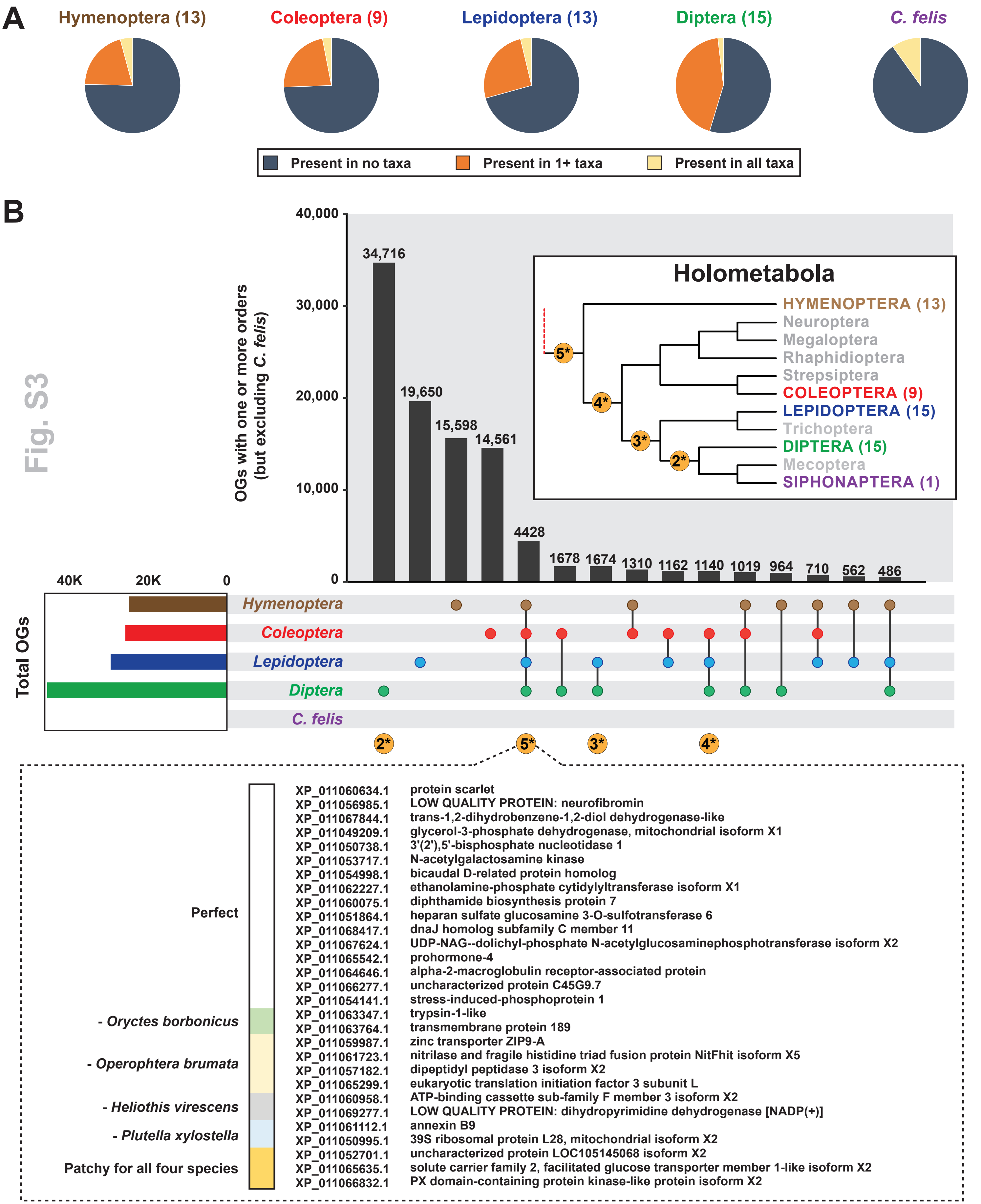
A
Hymenoptera (13)
Coleoptera (9)
Lepidoptera (13)
Diptera (15)
C. felis
Present in no taxa
Present in 1+ taxa
Present in all taxa
B
40,000
30,000
20,000
10,000
0
OGs with one or more orders (but excluding C. felis)
34,716
19,650
15,598
14,561
4428
1678
1674
1310
1162
1140
1019
964
710
562
486
Holometabola
HYMENOPTERA (13)
Neuroptera
Megaloptera
Rhaphidioptera
Strepsiptera
COLEOPTERA (9)
LEPIDOPTERA (15)
Trichoptera
DIPTERA (15)
Mecoptera
SIPHONAPTERA (1)
5*
4*
3*
2*
40K
20K
0
Total OGs
Hymenoptera
Coleoptera
Lepidoptera
Diptera
C. felis
2*
5*
3*
4*
XP_011060634.1 protein scarlet
XP_011056985.1 LOW QUALITY PROTEIN: neurofibromin
XP_011067844.1 trans-1,2-dihydrobenzene-1,2-diol dehydrogenase-like
XP_011049209.1 glycerol-3-phosphate dehydrogenase, mitochondrial isoform X1
XP_011050738.1 3'(2'),5'-bisphosphate nucleotidase 1
XP_011053717.1 N-acetylgalactosamine kinase
XP_011054998.1 bicaudal D-related protein homolog
XP_011062227.1 ethanolamine-phosphate cytidylyltransferase isoform X1
XP_011060075.1 diphthamide biosynthesis protein 7
XP_011051864.1 heparan sulfate glucosamine 3-O-sulfotransferase 6
XP_011068417.1 dnaJ homolog subfamily C member 11
XP_011067624.1 UDP-NAG--dolichyl-phosphate N-acetylglucosaminephosphotransferase isoform X2
XP_011065542.1 prohormone-4
XP_011064646.1 alpha-2-macroglobulin receptor-associated protein
XP_011066277.1 uncharacterized protein C45G9.7
XP_011054141.1 stress-induced-phosphoprotein 1
XP_011063347.1 trypsin-1-like
XP_011063764.1 transmembrane protein 189
XP_011059987.1 zinc transporter ZIP9-A
XP_011061723.1 nitrilase and fragile histidine triad fusion protein NitFhit isoform X5
XP_011057182.1 dipeptidyl peptidase 3 isoform X2
XP_011065299.1 eukaryotic translation initiation factor 3 subunit L
XP_011060958.1 ATP-binding cassette sub-family F member 3 isoform X2
XP_011069277.1 LOW QUALITY PROTEIN: dihydropyrimidine dehydrogenase [NADP(+)]
XP_011061112.1 annexin B9
XP_011050995.1 39S ribosomal protein L28, mitochondrial isoform X2
XP_011052701.1 uncharacterized protein LOC105145068 isoform X2
XP_011065635.1 solute carrier family 2, facilitated glucose transporter member 1-like isoform X2
XP_011066832.1 PX domain-containing protein kinase-like protein isoform X2
Perfect
- Oryctes borbonicus
- Operophtera brumata
- Heliothis virescens
- Plutella xylostella
Patchy for all four species
